# Nanoparticle Albumin‑Bound Paclitaxel and Solvent-Based Paclitaxel as Chemotherapy Options for Patients With Advanced Gastric Cancer: A Systematic Review and Meta-Analysis

**DOI:** 10.7759/cureus.41711

**Published:** 2023-07-11

**Authors:** Mahesh Gangannapalle, Husna Shahnoor, Lubna Sattar, Talwinder K Nagi, Marwah Al-Tekreeti, Muhammad Waqas Khan, Madiha D Haseeb, Areeba Khan

**Affiliations:** 1 Medicine, University of Perpetual Help System DALTA, Las Piñas, PHL; 2 Internal Medicine, Deccan College of Medical Sciences, Hyderabad, IND; 3 Medicine, Shadan Institute of Medical Sciences, Hyderabad, IND; 4 Internal Medicine, Florida Atlantic University Charles E. Schmidt College of Medicine, Boca Raton, USA; 5 Public Health, American Public University System, Charles Town, USA; 6 Medicine, Services Institute of Medical Sciences, Lahore, PAK; 7 Neurology, Dow University of Health Sciences, Karachi, PAK; 8 Critical Care Medicine, United Medical and Dental College, Karachi, PAK

**Keywords:** recurrent gastric cancer, progression-free survival, solvent-based paclitaxel, meta-analysis, overall survival, nanoparticle albumin‑bound paclitaxel

## Abstract

The aim of this study is to assess and compare the effectiveness and safety of nanoparticle albumin-bound paclitaxel (nab-PTX) and solvent-based PTX (sb-PTX) as treatment options for advanced gastric cancer. This meta-analysis was reported according to the Preferred Reporting Items for Systematic Review and Meta-Analysis (PRISMA) guidelines. We carried out a comprehensive search of PubMed, Google Scholar, and EMBASE from inception to June 15, 2023. The search strategy included the following keywords: "Nanoparticle albumin-bound paclitaxel," "solvent-based paclitaxel," and "advanced gastric cancer," along with their synonyms and medical subject heading (MeSH) terms. In this meta-analysis, the primary outcome was the comparison of overall survival and progression-free survival between the two groups. For safety purposes, we compared the risk of hematological and non-hematological events between the two groups. Four studies were included in this meta-analysis enrolling 1052 patients (483 received nb-PTX and 569 received sb-PTX). In terms of efficacy, nab-PTX showed favorable trends in overall survival and progression-free survival, despite no statistically significant differences being reported. The subgroup meta-analysis showed that nab-PTX seemed to have a better effect on peritoneal metastasis compared to sb-PTX. Regarding safety, the number of patients with neutropenia and leucopenia was significantly higher in the nab-PTX group compared to the sb-PTX group. However, the difference was statistically insignificant. Future research should focus on conducting more robust studies to further validate these findings and establish a stronger evidence base for the use of nab-PTX in this patient population.

## Introduction and background

Gastric cancer holds a significant position among cancers globally. It is the fifth most frequently detected cancer and stands as the third primary contributor to cancer-related deaths worldwide [1]. Approximately half of the patients are diagnosed with inoperable, locally advanced, or metastatic disease at the time of diagnosis. When dealing with advanced gastric cancer (AGC) that cannot be surgically removed or has recurred, systemic chemotherapy plays a vital role in alleviating symptoms and enhancing survival rates [2].

According to multiple guidelines, the preferred initial chemotherapy for unresectable advanced or recurrent gastric cancer (AGC) consists of a combination of fluoropyrimidine and platinum drugs [3,4]. The RAINBOW trial provided evidence that the weekly administration of paclitaxel (PTX) offers improved overall survival (OS) along with ramucirumab compared to weekly PTX alone in previously treated AGC patients [5]. As a result, PTX + RAM has now become the established standard second-line chemotherapy. Nanoparticle albumin-bound paclitaxel (nab-PTX) is a specialized form of paclitaxel that is bound to albumin particles, resulting in a solvent-free formulation with a particle size of 130 nm [6].

The mechanism of action of nab-PTX in the treatment of acute gastric cancer is primarily attributed to the PTX component of the formulation [7]. PTX, a microtubule-stabilizing agent, exerts its effects by binding to the β-tubulin subunit of microtubules, thereby promoting microtubule assembly and inhibiting their disassembly. This leads to the stabilization of microtubules and disruption of the normal dynamic equilibrium of microtubule structures within the cancer cells [8]. The stabilization of microtubules by nab-PTX results in the inhibition of mitotic spindle formation during cell division. This interference with mitosis prevents proper chromosome segregation and cell division, ultimately leading to cell cycle arrest and subsequent apoptosis (programmed cell death) of the cancer cells [9]. Unlike regular PTX administration, nab-PTX does not contain polyethoxylated castor oil or hydrated ethanol, reducing the risk of hypersensitivity reactions for patients [6]. Consequently, nab-PTX can be administered for a shorter duration without the need for premedication, making it a suitable option for patients with alcohol intolerance [6]. Therefore, in clinical practice, nab-PTX offers greater benefits and convenience compared to traditional sb-PTX administration.

The ABSOLUTE trial provided evidence that weekly administration of nab-PTX is not inferior to weekly solvent-based paclitaxel (sb-PTX) in terms of OS, with median OS values of 11.1 months and 10.9 months, respectively. The hazard ratio (HR) was 0.97, indicating comparable efficacy between the two treatments. The trial also indicated positive trends in progression-free survival (PFS) with median values of 5.3 months for nab-PTX and 3.8 months for sb-PTX, with an HR of 0.88. Additionally, there was an improvement in the overall response rate (ORR) with nab-PTX (33%) compared to sb-PTX (24%), although the difference was not statistically significant (p=0.10). These findings establish nab-PTX as a viable second-line chemotherapy option for AGC patients [6].

Not many studies have been conducted to directly compare the efficacy of nab-PTX and sb-PTX in patients with AGC. Consequently, to increase the power of the findings, we conducted a meta-analysis of available studies. The aim of this meta-analysis was to assess and compare the effectiveness and safety of nab-PTX and sb-PTX as treatment options for AGC.

## Review

Methodology

This meta-analysis was reported according to the Preferred Reporting Items for Systematic Review and Meta-Analysis (PRISMA) guidelines. We carried out a comprehensive search of PubMed, Google Scholar, and EMBASE from inception to June 15, 2023. The search strategy included the following keywords: "Nanoparticle albumin-bound paclitaxel," "solvent-based paclitaxel," and "advanced gastric cancer," along with their synonyms and medical subject heading (MeSH) terms. All published studies in all forms of publications were identified, irrespective of country, language, and year of publication. Additionally, the reference lists of all included studies were manually screened.

Study Selection

The selection criteria for inclusion in the meta-analysis included (a) studies that compared nab-PTX and sb-PTX in patients with AGC either alone or in combination with other drugs; (b) studies that included patients aged 18 years or older; and (c) studies that reported the required outcomes. We excluded studies without a comparison group. We also excluded case reviews, editorials, and review articles. Two investigators independently reviewed articles obtained through online database searching using abstracts and titles. Full texts of eligible studies were obtained, and a detailed evaluation was conducted based on pre-defined inclusion and exclusion criteria. Any disagreements between the two authors were resolved through consensus.

Data Extraction and Quality Assessment

Two reviewers independently extracted data from all included studies using an Excel Spreadsheet. The data extracted from the included studies included the name of the first author, publication year, region of the study, study design, groups, study dose, sample size, follow-up duration, study outcomes, and participants' characteristics. In this meta-analysis, the primary outcome was the comparison of overall survival and PFS between the two groups. For safety purposes, we compared the risk of hematological and non-hematological events between the two groups. Quality assessment was performed by two authors using the Newcastle-Ottawa scale and the Cochrane risk of bias tool for observational studies and randomized control trials (RCTs), respectively.

Statistical Analysis

The statistical analysis of the data was performed using Review Manager (RevMan) version 5.4.1 (The Cochrane Collaboration, London, United Kingdom). For comparisons, HRs were calculated along with 95% confidence intervals (CI) using a fixed-effect or random-effect model based on heterogeneity. For safety analysis, the risk ratio (RR) was calculated along with 95% CI. All P-values were two-tailed, and the level of statistical significance was set at 0.05 for all tests. The heterogeneity among the study results was presented as an I-square value. If the I-square value was less than 50%, a fixed-effect model was used. Otherwise, the analysis was conducted using a random-effect model. Subgroup analysis was performed based on different groups including age, metastasis, and number of sites involved.

Results

Table 1 shows the PRISMA flowchart of study selection. Online database searching yielded 544 studies. After removing duplicates, 522 studies were screened based on titles and abstracts. We obtained full texts of 12 studies and a thorough assessment was done based on pre-defined inclusion and exclusion criteria. Finally, four studies were included in this meta-analysis enrolling 1052 patients (483 received nb-PTX and 569 received sb-PTX). Table 1 shows the characteristics of included studies. Three studies were retrospective cohort studies, and one was an RCT. The pooled sample size of individual studies ranged from 128 to 483 patients. Table 2 shows quality assessment of included studies.

**Figure 1:**
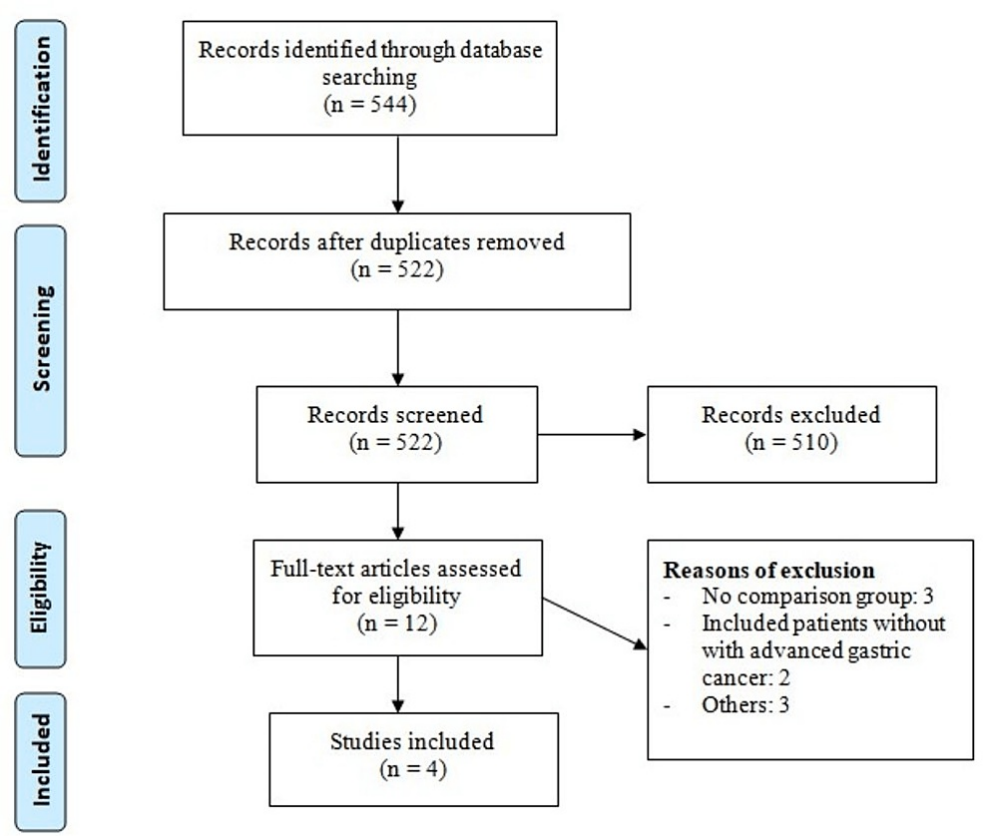
PRISMA flowchart PRISMA: Preferred Reporting Items for Systematic Review and Meta-Analysis

**Table 1:** Characteristics of included studies RCT: Randomized-control trial; nab-PTX: nanoparticle albumin-based paclitaxel; sb-PTX: solvent-based paclitaxel

Author Name	Year	Design	Groups	Sample Size	Dose	Follow-up	Age (Years)	Male (%)
Ishikawa et al. [2]	2020	Retrospective Cohort	nab-PTX	35	100 mg/m2 intravenously over 30 min on days 1, 8, and 15	30 Months	66.6 vs 67.2	74.2 vs 57.1
			sb-PTX	93	80 mg/m2 of PTX intravenously over 60 min on days 1, 8, and 15			
Okunaka et al. [10]	2020	Retrospective Cohort	nab-PTX	113	100 mg/m2 intravenously over 30 min on days 1, 8, and 15	42 Months	67 vs 69	69 vs 64
			sb-PTX	138	80 mg/m2 of PTX intravenously over 60 min on days 1, 8, and 15			
Nakasya et al. [11]	2022	Retrospective Cohort	nab-PTX	95	100 mg/m2 intravenously over 30 min on days 1, 8, and 15	30 Months	67 vs 69	73 vs 73
			sb-PTX	95	80 mg/m2 of PTX intravenously over 60 min on days 1, 8, and 15			
Shitara et al. [6]	2017	RCT	nab-PTX	240	100 mg/m2 intravenously over 30 min on days 1, 8, and 15	30 Months	67 vs 65	74 vs 72
			sb-PTX	243	80 mg/m2 of PTX intravenously over 60 min on days 1, 8, and 15			

**Table 2:** Quality assessment

Quality Assessment for Observational Studies					
Study ID	Selection	Comparibility	Outcome		
Ishikawa et al. [2]	3	2	3		
Nakasya et al. [10]	2	2	3		
Okunaka et al. [11]	4	2	3		
Quality Assessment for Randomized Control Trial					
Study ID	Selection	Performance	Attrition	Reporting	Other
Shitara et al. [6]	Low	Low	Low	Low	Unclear

Meta-Analysis of Outcomes

**Figure 2:**
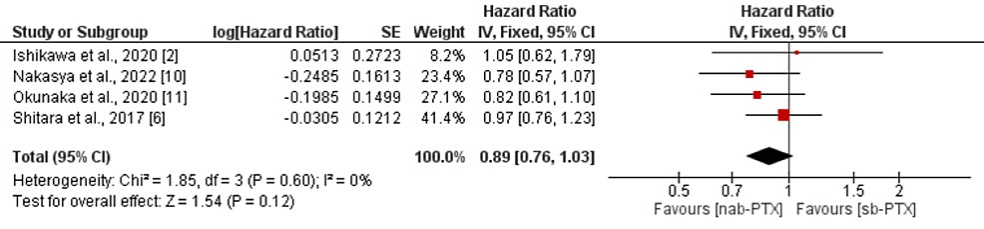
Overall survival nab-PTX: nanoparticle albumin-based paclitaxel; sb-PTX: solvent-based paclitaxel Source: References [2,6,10,11]

Overall survival: In total, four studies were included in the pooled analysis of overall survival between two groups. As shown in Figure 2, no significant difference was found between patients who received nab-PTX and paclitaxel in terms of overall survival (HR: 0.89, 95% CI: 0.76-1.03, p-value: 0.12). No significant heterogeneity was reported among the study results (I-square: 0%, p-value: 0.60).

**Figure 3:**
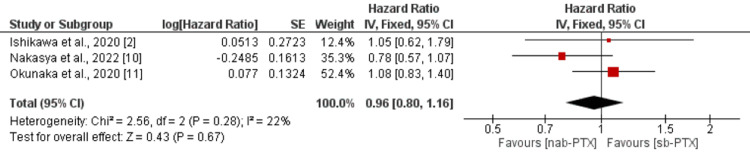
Progression-free survival nab-PTX: nanoparticle albumin-based paclitaxel; sb-PTX: solvent-based paclitaxel Source: References [2,10,11]

Progression-free survival: In total, three studies were included in the pooled analysis of PFS. Pooled analysis showed that no significant difference was found between patients who received nab-PTX and paclitaxel in relation to PFS (HR: 0.96, 95% CI: 0.80-1.16, p-value: 0.67) as shown in Figure 3. No significant heterogeneity was reported among the study results (I-square: 22%, p-value: 0.28).

Safety Analysis

Table 2 shows the results of safety analysis. When comparing the safety events of nab-PTX and sb-PTX, certain differences emerge. In terms of hematological side effects, nab-PTX exhibited higher rates of leucopenia and neutropenia (1.21; 95% CI: 1.04-1.42) compared to sb-PTX but the differences are statistically insignificant. Additionally, anemia was more prevalent with nab-PTX, showing a higher risk ratio of 1.30 (95% CI: 1.14-1.47). However, the occurrence of febrile neutropenia and gastric hemorrhage is insignificantly different between the two groups.

**Table 3:** Safety analysis *: Significant at p-value<0.05; CI: confidence interval

Safety Events	RR (95% CI)
Hematological	
Leucopenia	1.09 (0.99-1.21)
Neutropenia	1.15 (0.99-1.34)
Anemia	1.30 (1.14-1.47)*
Non-hematological	
Febrile Neutropenia	1.31 (0.66-2.58)
Gastric Hemmorhage	5.69 (0.77-42.16)

Subgroup Analysis

Table 3 shows the findings of subgroup analysis. Results demonstrated that nab-PTX was more favorable in patients with peritoneal metastasis and patients with age less than 65 years. No significant differences were reported between other subgroups.

**Table 4:** Subgroup analysis HR: Hazard ratio; CI: confidence interval

Outcomes	Groups	Progression-Free survival		Overall Survival	
		HR (95% CI)	P-value of subgroup differences	HR (95% CI)	P-value of Subgroup Differences
Peritoneal Metastasis	No	0.92 (0.77-1.65)	0.03	1.11 (0.75-1.83)	0.04
	Yes	0.70 (0.51-0.96)		0.85 (0.69-1.05)	
Duration of Prior Chemotherapy	< 6 Months	0.84 (0.63-1.11)	0.52	0.87 (0.65-1.17)	0.27
	>= 6 Months	0.95 (0.74-1.22)		1.09 (0.83-1.44)	
Age	>65 Years	0.57 (0.38-0.86)	0.05	0.86 (0.67-1.11)	0.31
	<=65 Years	0.95 (0.69-1.30)		1.03 (0.82-1.28)	
Number of Meta-static Sites	<2	0.85 (0.57-1.29)	0.69	1.00 (0.76-1.31)	0.59
	>=2	0.77 (0.56-1.06)		0.91 (0.73-1.13)	

Discussion

To our knowledge, this is the first meta-analysis comparing the efficacy and safety of nab-PTX and sb-PTX as chemotherapy options in patients with AGC. In terms of efficacy, nab-PTX showed favorable trends in overall survival and PFS, despite no statistically significant differences being reported. The study conducted by Katsaounis et al. demonstrated the efficacy of nab-PTX, even in the taxane-based pretreated group of patients [12]. The effectiveness of nab-PTX can be attributed to its capacity to utilize the albumin transport pathway mediated by gp60 and caveolae, allowing it to cross the endothelial lining of blood vessels and reach the tumor. Once inside the tumor, nab-PTX may preferentially accumulate and be retained due to the presence of tumoral SPARC [13].

The subgroup meta-analysis showed that nab-PTX seemed to have a better effect on peritoneal metastasis compared to sb-PTX. The potential effectiveness of nab-PTX and sb-PTX in treating peritoneal metastasis could be linked to variances in their drug compositions. Nanoparticle technology that employs human protein albumin is believed to utilize natural routes to target tumors more specifically and deliver higher quantities of the drug while also minimizing certain adverse effects associated with solvent-based formulations [14]. Albumin exhibits a strong attraction to hydrophobic drugs like PTX and is able to cross the endothelial barrier of blood vessels by binding to the gp60 albumin receptor and triggering caveolae-mediated endothelial transcytosis [15,16]. A preclinical investigation conducted on a rabbit model, where nab-PTX and sb-PTX were administered intraperitoneally, demonstrated that nab-PTX exhibited superior penetration through the peritoneum compared to sb-PTX. This enhanced penetration is likely primarily attributed to the improved solubility of the nab-PTX formulation [17]. As the sample size of the included studies is low, these findings need to be interpreted with caution. The ongoing P-SELECT trial, comparing nab-PTX+RAM versus PTX+RAM second-line chemotherapy for acute gastric cancer patients with peritoneal metastasis, may confirm these findings [18].

Regarding safety, the number of patients with neutropenia, anemia, and leucopenia was significantly higher in the nab-PTX group compared to the sb-PTX group. These hematological toxicities are considered manageable. A retrospective study found that patients who received weekly PTX experienced a significant decrease in neutrophil count, which was strongly correlated with improved efficacy [19]. Additionally, a prospective study indicated that patients who underwent dose-escalation of weekly PTX guided by neutropenia achieved better PFS rates compared to those who received the standard dose of weekly PTX [20].

The study has certain limitations. Firstly, only four studies were included in this meta-analysis, three of which were observational. Secondly, the sample size of the included studies was low. Therefore, these findings need to be interpreted with caution. In the future, more large-scale studies are required to compare the efficacy and safety of nab-PTX and sb-PTX in patients with AGC.

## Conclusions

In conclusion, this meta-analysis compared the efficacy and safety of nab-PTX and sb-PTX as chemotherapy options for patients with AGC. While no statistically significant differences were observed in OS and PFS between nab-PTX and sb-PTX, nab-PTX showed favorable trends in both outcomes. Subgroup analysis indicated that nab-PTX may have a better effect on peritoneal metastasis compared to sb-PTX. The safety analysis revealed that nab-PTX was associated with a higher risk of hematological side effects, such as leucopenia and neutropenia, compared to sb-PTX. Future research should focus on conducting more robust studies to further validate these findings and establish a stronger evidence base for the use of nab-PTX in this patient population.
